# Machine learning with persistent homology and chemical word embeddings improves prediction accuracy and interpretability in metal-organic frameworks

**DOI:** 10.1038/s41598-021-88027-8

**Published:** 2021-04-26

**Authors:** Aditi S. Krishnapriyan, Joseph Montoya, Maciej Haranczyk, Jens Hummelshøj, Dmitriy Morozov

**Affiliations:** 1 Computational Research Division, Lawrence Berkeley National Laboratory, Berkeley, CA 94720 USA; 2 Toyota Research Institute, Los Altos, CA 94022 USA; 3 IMDEA Materials Institute, C/Eric Kandel 2, 28906 Getafe, Madrid Spain

**Keywords:** Computational methods, Metal-organic frameworks, Porous materials, Applied mathematics

## Abstract

Machine learning has emerged as a powerful approach in materials discovery. Its major challenge is selecting features that create interpretable representations of materials, useful across multiple prediction tasks. We introduce an end-to-end machine learning model that automatically generates descriptors that capture a complex representation of a material’s structure and chemistry. This approach builds on computational topology techniques (namely, persistent homology) and word embeddings from natural language processing. It automatically encapsulates geometric and chemical information directly from the material system. We demonstrate our approach on multiple nanoporous metal–organic framework datasets by predicting methane and carbon dioxide adsorption across different conditions. Our results show considerable improvement in both accuracy and transferability across targets compared to models constructed from the commonly-used, manually-curated features, consistently achieving an average 25–30% decrease in root-mean-squared-deviation and an average increase of 40–50% in R^2^ scores. A key advantage of our approach is interpretability: Our model identifies the pores that correlate best to adsorption at different pressures, which contributes to understanding atomic-level structure–property relationships for materials design.

## Introduction

Metal–organic frameworks (MOFs) exhibit properties beneficial for a number of applications. Their porosity and large internal surface areas make them promising adsorbents for gas separation and storage; their diverse chemistry leads to their use as catalysts^1–3^. The number of MOF structures is massive—there are thousands of experimentally synthesized structures, but also many more hypothesized ones—creating a need for efficient tools and approaches to quickly identify MOFs best suited for a given applications.

The properties defining the best MOFs are dependent on the application. For example, different gas adsorptions have different applications: for example, adsorption of methane in the 65–5.8 bar range is relevant to on-board vehicular natural gas storage technologies^4^, while adsorption of carbon dioxide at lower pressure is important for CO_2_ capture from flue gases^5^.

Molecular simulations have played an important role in the prediction of adsorption and diffusion behaviour of guest species in nanoporous materials. They have allowed computation of Henry’s coefficients, adsorption loadings and diffusion coefficients at various conditions^6^. But a larger challenge remains: to advance our understanding of MOFs, it is necessary to recognize geometric and chemical features responsible for their performance in particular applications. These features offer useful clues for the design of new materials.

Machine learning offers a promising research direction to address this challenge. ML techniques^7,8^ have been used to screen large databases of MOFs, and to predict their properties faster than molecular simulations. Furthermore, feature representations developed for ML help identify correlations between MOF features and target properties. This makes it possible to relate input features to a MOF’s performance in a particular application. To do so effectively, one needs to find interpretable feature descriptors, whose values can be related to recognizable MOF properties^9–14^. Additionally, the diversity of properties and the vast number of structures makes it especially desirable to have an automatic framework to generate expressive features that work across multiple applications, enabling more transferability and less “handcrafting.”

Creating a universal representation from the input material structure, suitable for all different prediction tasks, is incredibly complicated. Typically, domain experts select specific features as the model input, usually tailored to making predictions about a particular property of interest. Often, this approach requires a large amount of manual processing to extract the necessary features^15^. For example, in the case of gas adsorption at high pressure, guest molecules tend to occupy the entire void space in a material, so void fraction can be used in predictive models. In contrast, for gas adsorption at low pressures, the guest molecules aggregate in the strongly binding regions of the material’s pore—standard structural descriptors are not able to capture this information as well. Additionally, chemical interactions of the system, in particular local strong adsorption sites, are important in determining some gas adsorption properties; this information also needs to be encoded in material descriptors.

Besides geometry and topology, chemical makeup of the internal surfaces is key for predicting MOF properties. Chemistry is especially important for predicting adsorption capacities at low pressures. Previous approaches have constructed chemical descriptors by incorporating information from MOF building blocks, such as functional groups^13,16,17^. These approaches have resulted in some improvements in predictive capabilities, but they still require manual feature curation to inspect all of the building blocks in the dataset. Moreover, the prediction accuracy of these descriptors often does not transfer across structures and properties.

In this paper, we describe how to overcome the above challenges and present an end-to-end ML framework that automatically generates a material representation, while only requiring the basic material structure (atomic coordinates and elemental composition) as input. As a consequence, this approach avoids handcrafting representations that do not transfer across property predictions. We use a topological descriptor, called persistent homology^18^, to compute multi-scale signatures of the channels and voids in the pores of the material. There have been previous approaches applying topological data analysis to materials^19,20^; however, in this work, we show that descriptors can be constructed from topological data analysis for downstream machine learning tasks for materials.

Additionally, we use features built using word embedding techniques^21^ to describe chemical information. As we demonstrate, this automated ML framework beats the standard structural descriptors in predicting a variety of materials properties. We also show that the overall methodology—coupling these features with ML algorithms that assign importances—opens the proverbial ML black box and allows us to interpret the predictions by identifying geometric and chemical properties relevant to different tasks.

## Methods

### Datasets

We demonstrate our approaches on three datasets corresponding to MOFs of various diversity, and across a range of CH_4_ and CO_2_ uptake pressures predicted using grand cannonical Monte Carlo simulations^22–24^. The first dataset is the hypothetical MOFs (hMOFs) database generated by Wilmer et al.^22^. The hMOF structures were taken from MOFDB (http://hmofs.northwestern.edu), which also has adsorption uptakes for carbon dioxide at five different pressures ranging from 0.05 bar to 2.5 bar.

The second dataset is the Boyd–Woo predicted MOF database^23^ with the predicted methane and carbon dioxide adsorption capacities at low and high pressure, and methane and carbon dioxide Henry’s coefficients. The Henry’s coefficients are expressed in terms of their logarithms.

Finally, we also included the 2019 CoREMOF dataset of the experimentally synthesized MOFs^24^.

For each structure in our dataset, as in our previous work^25^, we have determined the values of the following commonly–used geometric descriptors. We call these structural descriptors, and use them as a baseline to compare against topological descriptors:

- pore limiting diameter (PLD), in (Å), the diameter of the largest sphere to percolate through a material;
- largest cavity diameter (LCD), in (Å), the diameter of the largest sphere than can fit inside the material’s pore system;
- crystal density ($\rho$), in (kg/m^3^);
- accessible volume (AV), in (cm^3^/g);
- accessible surface area (ASA), in (m^2^/cm^3^).

The values for these descriptors were computed using the Zeo++ software package^26^.

### Automated topology–processing pipeline

**Figure 1:**
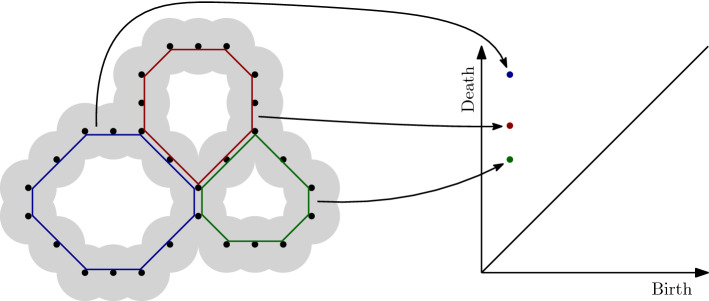
Schematic outlining point cloud to persistence diagram. (left) A point set (representing atomic centers) with balls of increasing radius around the points, (right) 1-dimensional persistence diagram of the point set. Representative cycles, corresponding to the points in the diagram, are highlighted with matching colors. The larger the loop, the higher the persistence value ($death-birth$). Figure created with Ipe 7.2.23 (http://ipe.otfried.org/).

We construct an automated pipeline to process an input MOF. We describe the topological structure of the MOFs using *persistent homology*^18^. To normalize the size of each MOF, expressed as (periodic) base cells of different sizes, we fill a (100 Å)^3^ cell with the atoms of the MOF. The size is chosen to be large enough to capture the statistics of the distribution of the topological features in every structure.

We represent a MOF as a union of hard spheres centered on its atoms. We increase the radii of these spheres and keep track of the changes in the topology of their union. The changes come in two types: a topological feature, such as a loop or a void, either appears or disappears. An important consequence of the algebraic formulation of this process is that these events can be paired uniquely, resulting in a set of birth–death pairs of radii, called a *persistence diagram*; see Fig. 1. There are two persistence diagrams relevant to us: a diagram that tracks births and deaths of loops that we interpret as tunnels in the MOF (we call these 1-dimensional features), and a diagram that tracks voids that we think of as pockets in the MOF (2-dimensional features). The difference in birth–death values is called *persistence* of the pair. Pairs of larger persistence capture more prominent pores in the MOF. We compute persistence diagrams using the Dionysus library (https://github.com/mrzv/dionysus).

Persistence diagrams are translated into vectors suitable as input for machine learning algorithms via a modification of *persistence images*, introduced by Adams et al.^27^. The birth–death pairs (*b*, *d*) are transformed into birth–persistence pairs $(b, d-b)$. They are then convolved with Gaussians and discretized onto a grid of a fixed size, by integrating the resulting mixture of Gaussians in the cells of the grid. For this, we use the resolution of $50 \times 50$ and a Gaussian spread of $\sigma = 0.15$.

### Word embeddings

We incorporate word embeddings of the chemical elements to represent a given MOF’s stoichiometric formula into our automated pipeline. We use this to capture the MOF’s chemical information. The chosen embeddings were constructed from a large corpus of abstracts with the word2vec algorithm^21^. The only input required is the elemental composition of the MOFs. Using word embeddings maintains the automated nature of our machine learning pipeline. While the use of word embeddings to featurize composition do represent an implicit knowledge that the chemical elements are distinct, they use no explicit element-specific properties and are themselves derived from an unsupervised learning procedure on raw text. From an input MOF structure, we construct features based on the composition of each MOF structure that represent word embeddings for the different elements in the MOF using the “matscholar_el” preset ElementProperty featurizer in matminer^28^. The features correspond to 200 embedding dimensions, with the minimum, maximum, range, mean, and standard deviation for each dimension, for a total of 1000 values. We note that the different datasets have different numbers of unique elements. For example, the hMOF dataset has eight, while the BW dataset has 16.

### Machine learning

We use random forest^29^ regression to predict carbon dioxide and methane adsorption uptakes at different pressures including infinite dilution (the Henry’s coefficients). One of our motivations for using the random forest is the ability to determine the feature importances in the model. The random forest algorithm builds an ensemble of decision trees and chooses a random subset of features for each one. The frequency with which a particular feature is chosen for a split is an estimate for the importance of the said feature.

We build trees for different groups of features: topological features, standard structural features, word embeddings, a combined model of topological features and word embeddings, a combined model of topological and structural features, and a combined model of topological features, structural features, and word embeddings. The topological features consist of both the 1D and 2D persistence images. We train the random forest on the specific target prediction of each material. Each of the forests consists of 500 trees, and the final prediction is the average of the prediction of all trees in the forest. After training the random forest on a training set, predictions are made on an unseen test set. For most of the predictions, we use an 80%/20% training-test split. The quality of the prediction is evaluated by comparing the predicted adsorption values and the correct adsorption values. We quantify our predictions by computing the root-mean-square deviation, $\sqrt{\sum ({\hat{y}}_i - y_i)^2 / n}$, and the coefficient of determination (R^2^), $1 - \sum (y_i - {\hat{y}}_i)^2) / \sum (y_i - {\bar{y}})^2$. We also note that there are other approaches to utilize persistence diagrams in machine learning algorithms, such as by directly processing the diagrams through an input persistence layer in a neural network^30^.

### Interpretability and representative cycles

The algorithm used to compute persistence^31^ tracks cycles that represent the topological features summarized in the persistence diagram. The cycles are not unique, but they reveal the atomic structures responsible for particular birth–death pairs. In a crystal structure, representative cycles correspond to channels or voids in the material. We visualize the cycles to better understand the topological features that appear in the MOFs. We choose which cycle to visualize using the feature importances found by the machine learning algorithms. We compute the representative cycles using the aforementioned Dionysus and visualize them with Zeo++ and VisIt.

## Results

We evaluate the accuracy of the automatically generated descriptors for our machine learning models by predicting a number of different targets across the different datasets. For each target, we calculate the root-mean-square deviation (RMSD) and coefficient of determination (R^2^ score). For each target and each dataset, we include results from models trained on only the topological features, only the word embeddings, and both the topological features and the word embeddings (T + WE). We also include results from the structural descriptors, described in Section “Datasets”, as a baseline. Finally, we incorporate the standard structural descriptors by including models combining topological and structural descriptors (T + S), as well as topological descriptors, structural descriptors, and word embeddings (T + S + WE).

### hMOF dataset

**Figure 2:**
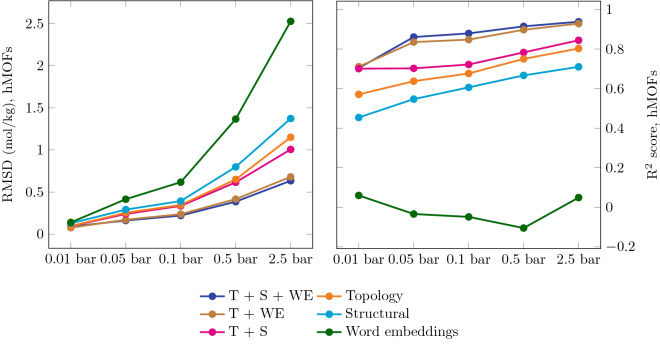
Model performances for hMOF dataset and CO_2_ adsorption. Comparison of root-mean-square deviation (left), coefficient of determination (right) in predicting gas uptakes in CO_2_ for different features at different pressures for the hMOF dataset. The RMSD is low at lower pressures because the distribution of carbon dioxide adsorption capacity has low variance in this regime. The topological features consistently outperform the standard structural features at all pressures. The T + WE and T + S + WE models achieve the best performance in general.

For the hMOF dataset, we predict carbon dioxide adsorption capacities at different pressures, as shown in Fig. 2. The RMSD is low at lower pressures because the distribution of carbon dioxide adsorption capacity has low variance in this regime. While the topology-based model outperforms the word embeddings, the model combining the two performs even better. We also see that the topological features always outperform the structural features, often significantly. The word embeddings do not perform as well here. This is likely due to the hMOF dataset lacking compositional diversity: the hMOF data set contains only eight unique elements. Nevertheless, word embeddings help boost the overall model performance when combined with the topological features.

We achieve the best performance by combining all three features together, but the accuracy achieved by subsets of the features is revealing. Adding structural to topological features slightly improves the performance, but doesn’t match that of all three features combined. On the other hand, the T + WE model performs only slightly worse than the T + S + WE model, indicating that the topological features capture most of the information that the structural features provide.

**Table 1:** Summary of model performances for hMOF dataset and CO_2_ adsorption.

Descriptor	0.01 bar	0.05 bar	0.1 bar	0.5 bar	2.5 bar
Structural	0.45	0.55	0.61	0.67	0.71
Topological	0.57	0.64	0.68	0.75	0.80
T + S	0.70	0.70	0.72	0.78	0.84
T + WE	**0.71**	0.84	0.85	0.90	0.93
T + S + WE	0.70	**0.86**	**0.88**	**0.92**	**0.94**
Best model, Fanourgakis et al.^11^	–	0.65	–	0.90	0.93

Machine learning results for carbon dioxide adsorption predictions on the hMOF dataset at different pressures, represented by R^2^ score. The best performing model for a given pressure is highlighted.

We compare our results to Fanourgakis et al.^11^, who used standard structural features and a customized featurization based on atom types to predict CO_2_ adsorption capacity in the hMOF dataset. Table 1 shows results for each of our models at different pressures, along with the best model from Fanourgakis et al^11^.

Our model does particularly well at low pressures, achieving an R^2^ score of 0.86 at 0.05 bar, compared to 0.65 from^11^. Carbon dioxide adsorption at low pressure has an important application: carbon capture from flue gases. Thus, it is particularly promising to have a generalized framework for accurate prediction of these targets. In general, our model transfers well across different pressures, as demonstrated by consistently high performance.

### BW dataset

We evaluate the accuracy of the automated machine learning pipeline on the BW dataset. We predict six targets grouped into three categories: the Henry’s coefficient (log(K$_{H}$)) for CO_2_ and CH_4_, the gas uptakes for CO_2_ at 0.15 and 16 bar, and the gas uptakes for CH_4_ at 5.8 and 65 bar.

**Table 2:** Model performance on BW dataset. Root-mean-square-deviation (RMSD) and coefficient of determination (R^2^ score) results in predicting the Henry’s coefficient (log k$_{H}$) for CO_2_ and CH_4_, gas uptakes for CO_2_, and gas uptakes for CH_4_ for the BW dataset.

Target	RMSD				R$^2$ score			
	S	T	T + WE	${{\Delta }}$	S	T	T + WE	${{\Delta }}$
log(K$_{H}$) CO$_2$	0.46	0.38	**0.33**	28.3%	0.60	0.68	**0.78**	30%
log(K$_{H}$) CH$_4$	0.27	0.20	**0.18**	33.3%	0.50	0.73	**0.79**	58%
0.15 bar CO$_2$	0.71	0.56	**0.49**	31%	0.57	0.71	**0.79**	38.6%
16 bar CO$_2$	1.9	2.53	**1.80**	5.3%	0.93	0.88	**0.94**	1.1%
5.8 bar CH$_4$	19.18	14.85	**13.97**	27.2%	0.68	0.82	**0.84**	23.5%
65 bar CH$_4$	23.87	20.61	**17.66**	26%	0.83	0.87	**0.90**	8.4%

Different sets of features (S = baseline structural, T = topological, T + WE = topological and word embeddings) are shown. For each target, the units are mol kg$^{-1}$ Pa$^{-1}$ and V$_{STP}$/V respectively. The best model is in bold. As the improvement from the topology + word embeddings is always greater than the structural features, the percentage of improvement (decrease in the case of RMSD and increase in the case of R$^2$ score) is also shown ($\Delta$).

Table 2 shows the results of these predictions for the BW dataset for the baseline structural features (S), topological features (T), and topological features + word embeddings (T + WE). As a general trend, the T + WE model outperforms the structural features by a large amount, with an average (across all targets) decrease of 25.2% in RMSD and an average increase of 26.6% in R$^2$ score. This is especially apparent for the Henry’s coefficient predictions and the CO$_2$ and CH$_4$ gas uptakes at low pressure. For these low pressure and infinite dilution gas adsorption predictions, to our knowledge, these topological descriptors are currently the best-performing descriptors that only take into account geometric information about the MOF. Supplementary Fig. 1 shows further visualization of the results with different sets of features.

### CoREMOF dataset

Finally, we evaluate the accuracy of the automated ML pipeline on the CoREMOF dataset. To narrow the dataset in a principled manner, we only include MOFs with a known topology net^32^, with each topology net appearing at least 15 times in the dataset for a total of approximately 50 topology nets in the whole dataset. We predict four targets here: the Henry’s coefficient (log(K$_{H}$)) for CO$_2$ and CH$_4$ and the gas uptakes for CH$_4$ at 5.8 and 65 bar.

**Table 3:** Model performance on CoREMOF dataset. Root-mean-square-deviation (RMSD) and coefficient of determination (R$^2$ score) results in predicting the Henry’s coefficient (log k$_{H}$) for CO$_{2}$ and CH$_{4}$ and gas uptakes for CH$_{4}$ for the CoREMOF dataset. Different sets of features (S = baseline structural, T = topological, T + WE = topological and word embeddings) are shown.

Target	RMSD				R$^2$ score			
	S	T	T + WE	${{\Delta }}$	S	T	T + WE	${{\Delta }}$
log(K$_{H}$) CO$_2$	0.90	0.73	**0.60**	33.3%	0.26	0.53	**0.69**	165%
log(K$_{H}$) CH$_4$	0.34	0.30	**0.24**	29.4%	0.55	0.65	**0.78**	41.2%
5.8 bar CH$_4$	27.15	22.00	**20.19**	25.7%	0.47	0.65	**0.71**	51.1%
65 bar CH$_4$	32.06	25.57	**24.57**	23.1%	0.76	0.85	**0.87**	14.5%

For each target, the units are mol kg$^{-1}$ Pa$^{-1}$ and V$_{STP}$/V respectively. The best model is in bold. As the improvement from the topology + word embeddings is always greater than the structural features, the percentage of improvement (decrease in the case of RMSD and increase in the case of R$^2$ score) is also shown ($\Delta$).

The improvement in using our ML framework in contrast to the commonly used structural features is particularly apparent in prediction improvement for the Henry’s coefficient’s of both CO$_2$ and CH$_4$ as well as low pressure CH$_4$. This improvement is especially noticeable in R$^2$ scores. For example, as seen in Table 3, our ML framework results in a 165% improvement over the structural features when predicting the Henry’s coefficient for CO$_2$. The implications here are vast as adsorption in the infinite dilution regime, such as is commonly seen at low partial pressures, is very important for carbon capture applications. Moreover, the same model provides additional improvement over RMSD and R$^2$ scores across all the targets, with an average decrease of 27.8% in RMSD and an average increase of 68% in R$^2$ score. While the same structural features cannot be used for accurate predictions across many different targets, in contrast, our model shows far greater transferability. Supplementary Fig. 2 shows further visualization of the results with different sets of features.

Notably, across all the datasets, the model combining topological and structural features only performs marginally better than the topological features alone. This indicates that the topological features are capturing almost everything the structural features capture, as well as much more.

## Interpretability

We also show the utility of our approach from an interpretability point of view. The feature importances extracted from the ML models contain important information to enhance our understanding of the material design process, and we explore multiple facets of this in the next sections.

### Feature analysis

The random forest algorithm infers the importance of individual features by measuring how frequently they are used by the decision trees to make a prediction about a MOF. In our methodology, there are three distinct types of features: topological, structural, and word embeddings. Further, topological features come in two types, 1-dimensional features that capture the distribution of channels in the MOF and 2-dimensional features that describe the voids. Each of those consists of 2500 individual features (pixels in the persistence image), but we combine them to infer the aggregate importance of the different feature types. In this section, we analyze contributions from the topological and word embedding features, since the structural features contribute little extra information.

**Figure 3:**
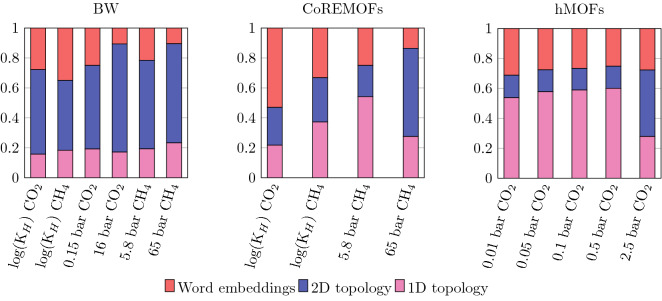
Feature analysis of machine learning models. Summary of relative feature importance across different targets for the 1D, 2D topological features, and word embeddings. The BW, CoREMOF, and hMOF datasets are shown here.

Figure 3 shows the relative importance of topological descriptors and word embeddings. For the BW dataset, 2D features are most important for the prediction, with word embeddings playing a larger role in the predictions of the Henry’s coefficient. For the CoREMOF dataset, word embeddings are more important, especially for the CO$_2$ Henry’s coefficient where they account for 50–60% of the decisions, with topological features dominating the importance of predictions for both low and high pressure methane adsorption (albeit, 1D features play a larger role in low pressure methane adsorption, while 2D features play a larger role in high pressure methane adsorption). For the hMOF dataset, 1D topological features are most important at low pressures, with 2D being more important at higher pressure, and word embeddings used in $\sim 30\%$ of the decisions.

As Fig. 3 shows, topological features play a major role in predicting gas adsorption, with the 1-dimensional channels being especially important for adsorption at low pressures in the CoREMOF and hMOF datasets, and 2-dimensions voids being important for the predictions with the BW dataset. The differences in feature importances can also be linked back to the data: for example, the CoREMOF MOFs tend to have smaller pores than the BW MOFs.

These results reveal the importance of different properties for different tasks. They support the claim that chemical information is more important for infinite dilution and low-pressure CO$_2$ adsorption. In these conditions, the specific interactions between the gas and the MOF framework, e.g. manifested as strong binding sites, play an important role in adsorption capacity—the word embeddings capture this non-structural information. On the other hand, methane adsorption at higher pressure is mostly described by 2D topology features, which can described voids at large, a trend that we also observed in zeolites^25^.

Our results also suggest why the conventional structural descriptors perform especially poorly when predicting CO$_2$ adsorption in hMOFs at low pressure or in the infinite dilution region. The standard structural features describe the pore geometry by the largest sphere to percolate through the materials and the largest sphere that can fit inside its pore system. At low pressures and/or in the infinite dilution region, the standard structural features are not able to capture the nuance of the gas molecules aggregating closer to the binding regions of the porous framework. In contrast, topological features record the widths of the channels that criss-cross the MOF as well as the sizes of different cavities. They also distinguishing between the distribution of channels and voids, by separating 1D and 2D topological features, and record other finer information about their shape.

### Topological features and representative cycles

Nanoporous materials, and especially MOFs, are known for how tunable they are: experimentalists can synthesize materials with precisely sized pores. Understanding how structure features influence a particular material property helps guide this process. Our approach incorporating persistent homology is especially helpful in this task.

The points in a persistence diagram correspond to voids and channels of specific sizes. A point (*b*, *d*) in a 2-dimensional diagram is generated by a cavity that can fit the largest sphere of radius *d*; the largest sphere that can access the cavity has radius *b*. A point (*b*, *d*) in a 1-dimensional diagram is produced by a channel in the material, specifically, by its narrowest “bottleneck.” The death value, *d*, records the radius of the largest sphere that can pass through this bottleneck. The birth value, *b*, records how close the atoms of the bottleneck are to each other.

For each dataset and each target property, the most important 1D and 2D birth-death points, as identified by the random forest algorithm, are listed in Table 4. We note a few patterns. In the case of methane adsorption in all three regimes (infinite dilution, low pressure, and high pressure), the 2D birth and death values are similar for both the BW and CoREMOF datasets—in fact, almost identical for the infinite dilution and high pressure cases. Specifically, birth values are around 2.3–2.4 Å for high pressure methane adsorption, and 3.4–3.8 Å for low pressure and infinite dilution methane adsorption. Death values are 3.2 Å for high pressure methane adsorption, and 4–4.6 Å for low pressure and infinite dilution methane adsorption. The radius of a methane molecule is assumed to be 3.8 Å. These results suggest that pores somewhat larger than this radius adsorb well at low pressures and partial pressures, while at high pressures slightly smaller pores influence the overall adsorption capacity of the MOF.

Another pattern to note in the hMOF dataset is that 1D death values get larger as pressure increases, meaning the size of the largest sphere able to pass through the channel increases. The radius of a CO$_2$ molecule is assumed to be 3.3 Å. For high pressure targets, the model picks out the channels that can accommodate the molecule of this size. The 1D birth/death values for lower pressures correspond to smaller pores, such as the porous surface, which is related to the binding regions of the material’s pore.

**Table 4:** Most important 1D/2D birth–death points for the different datasets (in Angstroms). These values correspond to the porous framework sizes most important for a given adsorption task.

Target property	1D birth	1D death	2D birth	2D death
**(a) BW dataset**				
log(K$_{H}$) CO$_{2}$	1	4	3.3	4.1
log(K$_{H}$) CH$_{4}$	1.6	2	3.6	4.4
0.15 bar CO$_{2}$	3.5	3.6	3.4	4
16 bar CO$_{2}$	1.7	2	3.1	3.9
5.8 bar CH$_{4}$	1.4	3	3.8	4.6
65 bar CH$_{4}$	3.6	4.3	2.3	3.2
**(b) CoREMOF dataset**				
log(K$_{H}$) CO$_{2}$	0.3	1.3	2.3	3.1
log(K$_{H}$) CH$_{4}$	0.3	1	3.6	4.4
5.8 bar CH$_{4}$	1	3.3	3.4	4
65 bar CH$_{4}$	3.9	4.8	2.4	3.2
**(c) hMOF dataset**				
0.01 bar CO$_{2}$	0.02	0.7	3.2	3.5
0.05 bar CO$_{2}$	1.1	1.6	1.6	2.1
0.1 bar CO$_{2}$	1.1	2.7	4.4	5.5
0.5 bar CO$_{2}$	1.3	3.5	4.7	5.8
2.5 bar CO$_{2}$	1	3.7	4	5.1

We can dissect topological representations further and extract representative cycles for each point. Although these cycles are not unique—we are at the mercy of certain choices persistent homology calculation makes—they are helpful in visualizing the cavities and channel bottlenecks represented by the points in the persistence diagram.

Since we train our machine learning algorithm on vectorized persistence images, we have to take an extra step to identify the points in a persistence diagrams with relevant representative cycles. We illustrate our steps for this approach in Supplementary Fig. 3.

**Figure 4:**
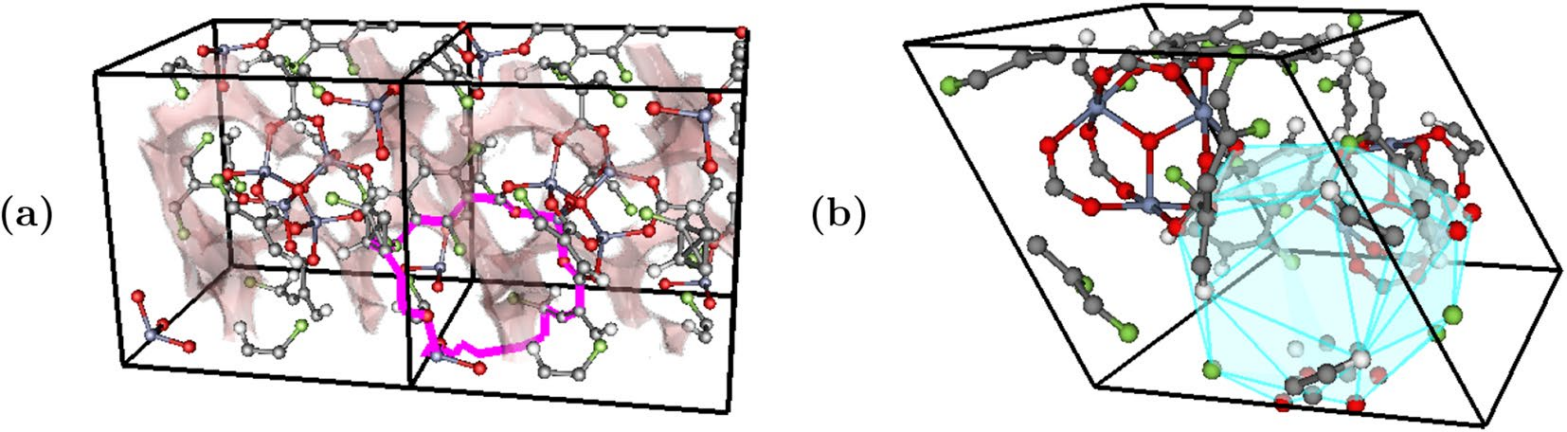
Example 1D and 2D representative cycles for different MOFs. (**a**) 1D channel, hMOF-675 (hMOFs) (**b**) 2D void, str-m4-o14-o14-acs-sym-5 (BW). The representative cycles are picked based on the approach described in Supplementary Fig. 3. Figure created with VisIt 3.1.4 (https://wci.llnl.gov/simulation/computer-codes/visit).

We extracted the representative cycles from the high gas adsorption MOFs from different databases. Two examples, including both 1D topology (channels) and 2D topology (voids), appear in Fig. 4. One notable trend is that the loop in Figure 4a is present in many of the materials in the hMOF dataset that have high CO_2_ adsorption at low pressure. Similarly, the void size seen in Fig. 4b is present in many of the MOFs with high Henry’s coefficients for CO_2_ adsorption.

**Figure 5:**
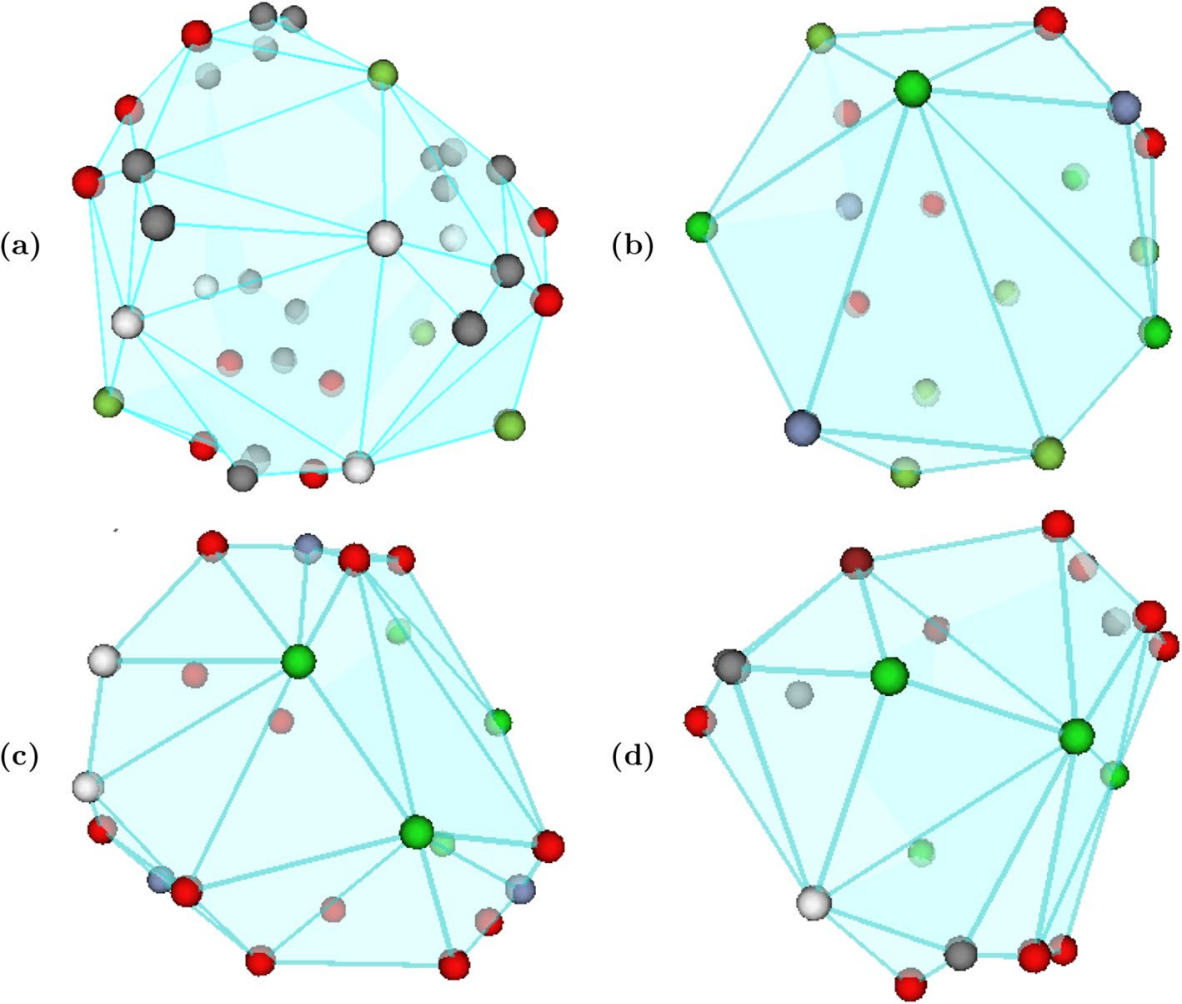
Correlating void structure to MOF property. (**a**) str-m4-o14-acs-sym-8 (**b**) str-m4-o1-o22-acs-sym-94 (**c**) str-m4-o1-o24-acs-sym-96 (**d**) str-m4-o1-o24-acs-sym-165. The representative cycles of voids corresponding to the void most correlated with the CO$_2$ Henry’s coefficient in example MOFs with high CO$_2$ Henry’s coefficients. The voids are all composed of a similar bonding structure, with each different atom type represented by a different color. As noted in^33^, the process of identifying the void structure that appears in top performing MOFs can be extremely time-consuming via manually detected features. Thus, we hope that our much faster and topologically—grounded approach will allow for further study in pinpointing the channel and void shapes and bonding structures that correlate best to important material’s properties, thereby encouraging the targeted design of structures to maximize desirable properties. Figure created with VisIt 3.1.4 (https://wci.llnl.gov/simulation/computer-codes/visit).

We expand on the latter by showing, as an example, in Fig. 5 the extracted voids that appear in a number of the top MOFs with a high CO_2_ Henry’s coefficient. As noted in^33^, the process of identifying the void structure that appears in top performing MOFs can be extremely time-consuming via manually detected features. Thus, we hope that our approach will allow for further study in pinpointing the channel and void shapes and bonding structures that correlate best to important material’s properties, thereby encouraging the targeted design of structures to maximize desirable properties.

### Word embeddings and material properties

We explore the interpretability of the word embeddings by relating their importances in predicting MOF properties and in predicting chemical properties of individual elements. The former we obtain from the random forests just as the importances of the topological features. To calculate the importances for individual elements, we retrieve word embeddings for all the elements in the matscholar database^21^ and use these as features to train models to predict various chemical properties—electronegativity, atomic radius, electrical resistivity, melting point, etc.–of the pure elements contained in pymatgen’s ‘periodic_table’ module^34^. We extract the feature importances for each of these models. Because each MOF has 1000 features, summarizing the distribution of 200 features over its elements, as described in Section “Word embeddings”, we sum up the MOF feature importances corresponding to the same elemental feature.

We take the subset of feature importances that account for 90% of the random forest decisions. By definition, these features describe the subspace of our input where most of the decisions are made to make a prediction about the given target property. Given a MOF target property and a chemical target property, we compute the Jaccard similarity between the two subsets of features. This metric measures the relative size of the subspace, important for the random forest decisions for both targets.

Table 5 lists the top three materials properties by similarity to each MOF target property; all of them have a Jaccard similarity greater than 0.4. Following this procedure, we identify the chemical property with the strongest informational relevance to a given MOF target property.

**Table 5:** Material properties sharing overlap with word embedding feature importances. Machine learning models trained with elemental word embeddings and materials properties are compared to the models trained with MOF composition word embeddings and MOF target properties for the CoREMOF dataset. The feature importances of each model are analyzed, and compared by Jaccard similarity. The top three materials properties most similar to the model trained to MOF target properties are listed.

Target property	1	2	3
log(K$_{H}$) CO$_{2}$	Electronegativity	Poisson’s ratio	Mendeleev’s number
log(K$_{H}$) CH$_{4}$	Electronegativity	Poisson’s ratio	Thermal conductivity
5.8 bar CH$_{4}$	Thermal conductivity	Poisson’s ratio	Brinell’s hardness
65 bar CH$_{4}$	Thermal conductivity	Electronegativity	Melting point

We focus on interpreting the results from a MOF design perspective. The word-embedding features play a bigger role than topology in predicting log(K$_{H}$) CO$_{2}$. For this target, the machine learning model trained on electronegativity was the most similar to the model trained on the word embeddings for each MOF. This suggests that local interactions are more significant in carbon dioxide adsorption in the infinite dilution regime, which is consistent with qualitative descriptions of low pressure or dilute-limit profiles of absorptivity in porous materials from literature^12^.

Thermal conductivity also appears multiple times, and is the most relevant elemental property for high pressure $\hbox {CH}_{4}$ adsorption. The relevance of thermal conductivity at higher pressures is more difficult to interpret, given that thermal conductivity contains an electronic and vibrational component. However, a relationship between thermal conductivity and MOF geometry has been suggested previously. Specifically, thermal conductivity correlates with pore size and porosity^35,36^, which in turn affects adsorption. Thus, when designing a MOF, including or substituting metal atoms which have low thermal conductivity in their phase pure form may improve adsorption in MOF structures. The coordination environment and identity of the coordinating linkers also likely plays a role in determining the trend for a given site. For reference, we have included the compositions of the high adsorption MOFs for each prediction task in the Supplementary Material.

Another materials property that appeared multiple times for multiple MOF targets was the Poisson’s ratio, which reflects elasticity of a material. This is another property that fits in the existing paradigm of MOF design: namely, flexibility. MOFs with flexible frameworks often are better adsorbents^37^, since they can accommodate a larger space to fit a gas molecule with less stress.

In summary, the latent information contained in the word embeddings overlaps with known descriptors for MOF gas adsorption, pointing to important chemical features for designing high adsorption MOFs^38^.

## Conclusions

We have developed an automated end-to-end machine learning framework for MOFs, and nanoporous materials in general, by using persistent homology and word embeddings. Our approach builds a complex and holistic representation of the materials using only the basic input material structure, requiring less handcrafting and domain expert guidance than the currently widely—used porosity and chemical descriptors. Our topological representation is a vectorized persistence diagram, obtained from the atomic coordinates of the normalized supercell representation of a materials’ crystal structure. It can be used in any machine learning algorithm. We augment the topological information with element embeddings, constructed from a large set of scientific abstracts via the word2vec algorithm^21^. They provide a generalized representation of the MOF composition. We have tested this approach on three different datasets, predicting several important methane and carbon capture adsorption targets at various pressures. These experiments show a significantly improved performance compared to standard structural descriptors. The topological features we compute are generic and transferable across different property targets. As the topological descriptors consistently outperform standard structural descriptors, they provide a simple way to boost the performance of any machine learning algorithm. Additionally, to our knowledge, these descriptors are the best purely geometric descriptors for predicting gas adsorption at low pressures and in the infinite dilution regime. Moreover, these descriptors are interpretable: their components can be traced to specific channels and voids in the crystal structure, which contributes to a greater understanding of structure–property relationships in MOFs.

We conclude by highlighting the key strengths of our approach.

1. It is an ML pipeline that can automatically generate descriptors for a particular material’s prediction task without the need to handcraft specific features. We make large gains in performance (ranging from an average 25–30% in root-mean-square-deviation and an average 45–50% increase in R^2^ scores) across numerous different gas adsorption targets.
2. The generalizability and transferability of our ML model provides a way to quickly screen any dataset to find the top MOFs for a particular task without the need to handcraft specific features, speeding up high-throughput screening of materials for adsorption applications. As our results show, topological descriptors should be used for any porous materials adsorption prediction task and bring us closer to having a universal predictor for adsorption in porous materials.
3. Our model helps guide materials design by directly connecting property predictions to the crystal structure, thereby encouraging the targeted design of structures to maximize desirable properties.

## Supplementary Information

Supplementary Information.

## Data Availability

The code for generation of the material representations is available at: http://www.github.com/a1k12/molecule-tda.

## References

[1] Jesse, L. C., Rowsell, E. C. Spencer, J. E., Howard, J. A. K. & Yaghi, O. M. Gas adsorption sites in a large-pore metal–organic framework. *Science*, **309**(5739):1350–1354, (2005). 10.1126/science.1113247 16123294

[2] Li J-R, Sculley J, Zhou H-C (2012). Metal–organic frameworks for separations. Chem. Rev..

[3] Yang D, Gates BC (2019). Catalysis by metal organic frameworks: Perspective and suggestions for future research. ACS Catal..

[4] He Y, Zhou W, Qian G, Chen B (2014). Methane storage in metal-organic frameworks. Chem. Soc. Rev..

[5] Sumida K, Rogow DL, Mason JA, McDonald T M, Bloch ED, Herm Z R, Bae T-H, Long JR (2012). Carbon dioxide capture in metal-organic frameworks. Chem. Rev..

[6] Odoh SO, Cramer CJ, Truhlar DG, Gagliardi L (2015). Quantum-chemical characterization of the properties and reactivities of metal-organic frameworks. Chem. Rev..

[7] Jablonka, K. M., Ongari, D., Moosavi, S. M., & Smit, B. Big-data science in porous materials: Materials genomics and machine learning. *Chem. Rev.*, **120**(16), 8066–8129 (2020). 10.1021/acs.chemrev.0c00004 PMC7453404 32520531

[8] Chong S, Lee S, Kim B, Kim J (2020). Applications of machine learning in metal-organic frameworks. Coord. Chem. Revi..

[9] Fernandez M, Barnard AS (2016). Geometrical properties can predict co2 and n2 adsorption performance of metal-organic frameworks (mofs) at low pressure. ACS Comb. Sci..

[10] Pardakhti M, Moharreri E, Wanik D, Suib SL, Srivastava R (2017). Machine learning using combined structural and chemical descriptors for prediction of methane adsorption performance of metal organic frameworks (mofs). ACS Comb. Sci..

[11] Fanourgakis GS, Gkagkas K, Tylianakis E, Froudakis GE (2020). A universal machine learning algorithm for large-scale screening of materials. J. Am. Chem. Soc..

[12] Moosavi, S. M., Nandy, A., Jablonka, K. M., Ongari, D., Janet, J. P., Boyd, P. G., Lee, Y., Smit, B., & Kulik, H. J. Understanding the diversity of the metal–organic framework ecosystem. *Nat. Commun.*, **11**(1), 4068 (2020). 10.1038/s41467-020-17755-8 PMC7426948 32792486

[13] Anderson R, Rodgers J, Argueta E, Biong A, Gómez-Gualdrón DA (2018). Role of pore chemistry and topology in the co2 capture capabilities of mofs: From molecular simulation to machine learning. Chem. Mater..

[14] Shi Z, Liang H, Yang W, Liu J, Liu Z, Qiao Z (2020). Machine learning and in silico discovery of metal-organic frameworks: Methanol as a working fluid in adsorption-driven heat pumps and chillers. Chem. Eng. Sci..

[15] Simon, C. M., Mercado, R., Schnell, S. K., Smit, B., & Haranczyk, M. What are the best materials to separate a xenon/krypton mixture? *Chem. Mater.*, **27**(12), 4459–4475 (2015).

[16] Borboudakis, G., Stergiannakos, T., Frysali, M., Klontzas, E., Tsamardinos, I., & Froudakis, G. E. Chemically intuited, large-scale screening of MOFs by machine learning techniques. *NPJ Comput. Mater.*, **3**(1), 40 (2017).

[17] Anderson, R., Biong, A., & Gómez-Gualdrón, D. A. Adsorption isotherm predictions for multiple molecules in mofs using the same deep learning model. *J. Chem. Theory Comput.*, **16**(2), 1271–1283 (2020). 10.1021/acs.jctc.9b00940 31922755

[18] Edelsbrunner H, Harer J (2007). Persistent homology: A survey. Contemp. Math..

[19] Lee, Y., Barthel, S. D., Dłotko, P., Moosavi, S. M., Hess, K. & Smit, B. Quantifying similarity of pore-geometry in nanoporous materials. *Nat. Commun.*, **8**(1), 15396 (2017). 10.1038/ncomms15396 PMC5457500 28534490

[20] Sørensen, S. S., Biscio, C. A. N., Bauchy, M., Fajstrup, L., & Smedskjaer, M. M. Revealing hidden medium-range order in amorphous materials using topological data analysis. *Sci. Adv.*, **6**(37), eabc2320 (2020). 10.1126/sciadv.abc2320 PMC11206462 32917687

[21] Tshitoyan V (2019). Unsupervised word embeddings capture latent knowledge from materials science literature. Nature.

[22] Wilmer, C. E. *et al*. Large-scale screening of hypothetical metal–organic frameworks. *Nat. Chem.*, **4**(2), 83–89 (2012). 10.1038/nchem.1192 22270624

[23] Boyd, P. G. & Woo Tom, K. A generalized method for constructing hypothetical nanoporous materials of any net topology from graph theory. *CrystEngComm*, **18**(21), 3777–3792 (2016).

[24] Chung, Y. G., *et al.* Advances, updates, and analytics for the computation-ready, experimental metal–organic framework database: Core mof 2019. *J. Chem. Eng. Data*, **64**(12), 5985–5998 (2019).

[25] Krishnapriyan AS, Haranczyk M, Morozov D (2020). Topological descriptors help predict guest adsorption in nanoporous materials. J. Phys. Chem. C.

[26] Willems TF, Rycroft CH, Kazi M, Meza JC, Haranczyk M (2012). Algorithms and tools for high-throughput geometry-based analysis of crystalline porous materials. Microporous and Mesoporous Mater..

[27] Adams H, Emerson T, Kirby M, Neville R, Peterson C, Shipman P, Chepushtanova S, Hanson E, Motta F, Ziegelmeier L (2017). Persistence images: A stable vector representation of persistent homology. J. Mach. Learn. Res.

[28] Ward L (2018). An open source toolkit for materials data mining. Comput. Mater. Sci..

[29] Breiman L (1995). Random forests. Int. J. Mach. Learn. Cybern..

[30] Swenson, N., Krishnapriyan, A. S., Buluc, A., Morozov, D., & Yelick, K. Persgnn: Applying topological data analysis and geometric deep learning to structure-based protein function prediction. arXiv:2010.16027 (2020).

[31] Edelsbrunner H, Letscher D, Zomorodian A (2002). Topological persistence and simplification. Discret. Comput. Geom..

[32] Li, M., Li, D., O'Keeffe, M., & Yaghi, O. M. Topological analysis of metal–organic frameworks with polytopic linkers and/or multiple building units and the minimal transitivity principle. *Chem. Rev.*, **114**(2), 1343–1370 (2014). 10.1021/cr400392k 24191753

[33] Martin, R. L. * et al*. Similarity-driven discovery of zeolite materials for adsorption-based separations. *ChemPhysChem*, **13**(16), 3595–3597 (2012). 10.1002/cphc.201200554 22915542

[34] Ong, S. P.* et al*. Python materials genomics (pymatgen): A robust, open-source python library for materials analysis. *Comput. Mater. Sci.*, **68**, 314–319 (2013).

[35] Sumirat, I., Ando, Y., & Shimamura, S. Theoretical consideration of the effect of porosity on thermal conductivity of porous materials. *J. Porous Mater.*, **13**(3), 439–443 (2006).

[36] Babaei, H., McGaughey, A. J. H., & Wilmer, C. E. Effect of pore size and shape on the thermal conductivity of metal–organic frameworks. *Chem. Sci.*, **8**, 583–589, (2017). 10.1039/c6sc03704f PMC5358541 28451205

[37] Coudert FX (2015). Responsive metal-organic frameworks and framework materials: Under pressure, taking the heat, in the spotlight, with friends. Chem. Mater..

[38] Lee, T., Chang, Y. H., & Lee, H. L. Crystallization process development of metal–organic frameworks by linking secondary building units, lattice nucleation and luminescence: insight into reproducibility. *CrystEngComm*, **19**(3), 426–441 (2017).

