# Supplementary material for: Machine learning with persistent homology and chemical word embeddings improves prediction accuracy and interpretability in metal-organic frameworks
Source: Sci Rep. 2021 Apr 26;11:8888. doi: 10.1038/s41598-021-88027-8 (PMC8076181; doi:10.1038/s41598-021-88027-8)
Supplement: Supplementary file 1 — Supplementary Information. [file 41598_2021_88027_MOESM1_ESM.pdf]

Supplementary Information

Aditi S. Krishnapriyan*[1, 2], Joseph Montoya[2], Maciej Haranczyk[3], Jens Hummelshøj[2], and Dmitriy Morozov[1]

[1]Computational Research Division, Lawrence Berkeley National Laboratory, Berkeley, California 94720, United States of America
[2]Toyota Research Institute, Los Altos, California 94022, United States of America
[3]IMDEA Materials Institute, C/Eric Kandel 2, 28906 Getafe, Madrid, Spain

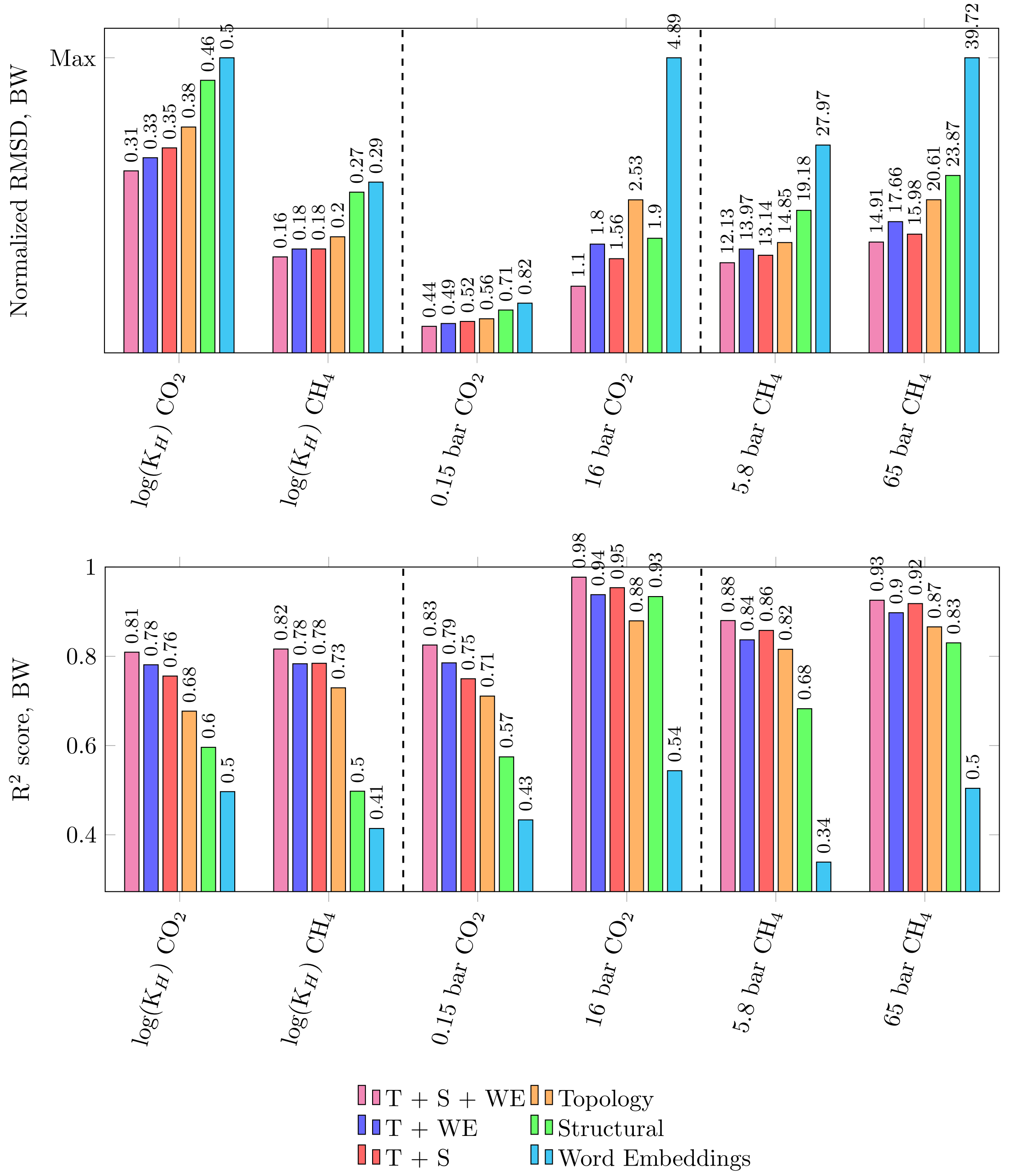

Figure 1: **Model performances on BW dataset.** Comparison of root-mean-square deviation (left), coefficient of determination (right) in predicting the Henry's coefficient (log k_H) for CO_2 and CH_4, gas uptakes for CO_2, and gas uptakes for CH_4, for different features for the BW20K dataset. For each target, the units are mol kg^{-1} Pa^{-1}, mmol/g, and VSTP/V respectively. Due to the difference in units between targets, RMSD values are normalized with respect to the maximum value in each category. The black, dashed line defines the categories that share the same units. In the case of RMSD, these categories also share a normalization factor. The actual RMSD and R^2 values are shown above each bar.

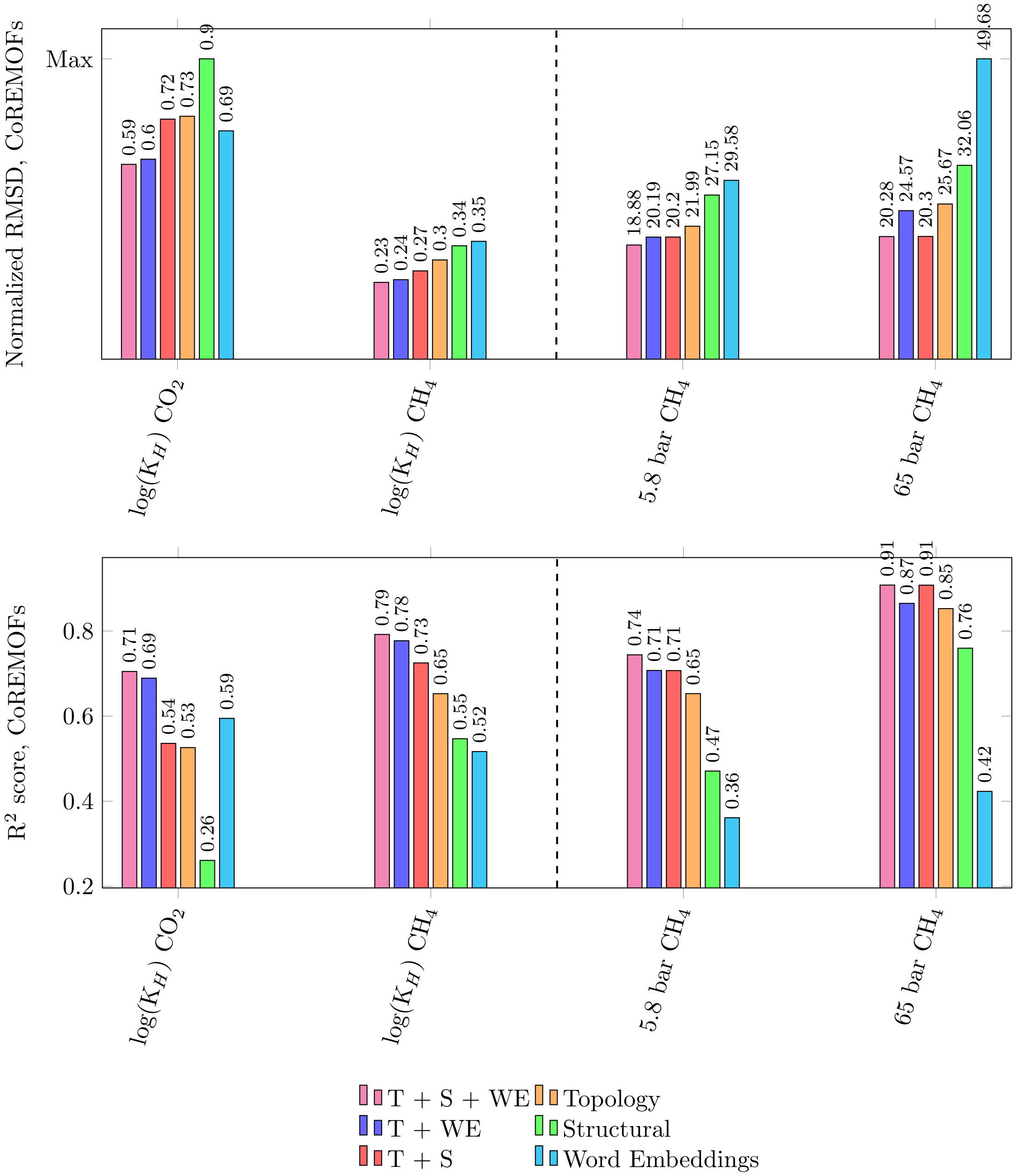

Figure 2: **Model performances on CoREMOF dataset.** Comparison of root-mean-square deviation (left), coefficient of determination (right) in predicting the Henry's coefficient(log k_H) for CO_2 and CH_4 and gas uptakes for CH_4, for different features for the CoREMOF dataset. For each target, the units are mol kg^{-1} Pa^{-1} and VSTP/V respectively. Due to the difference in units between targets, RMSD values are normalized with respect to the maximum value in each category. The black, dashed line defines the categories that share the same units. In the case of RMSD, these categories also share a normalization factor. The actual RMSD and R^2 values are shown above each bar.

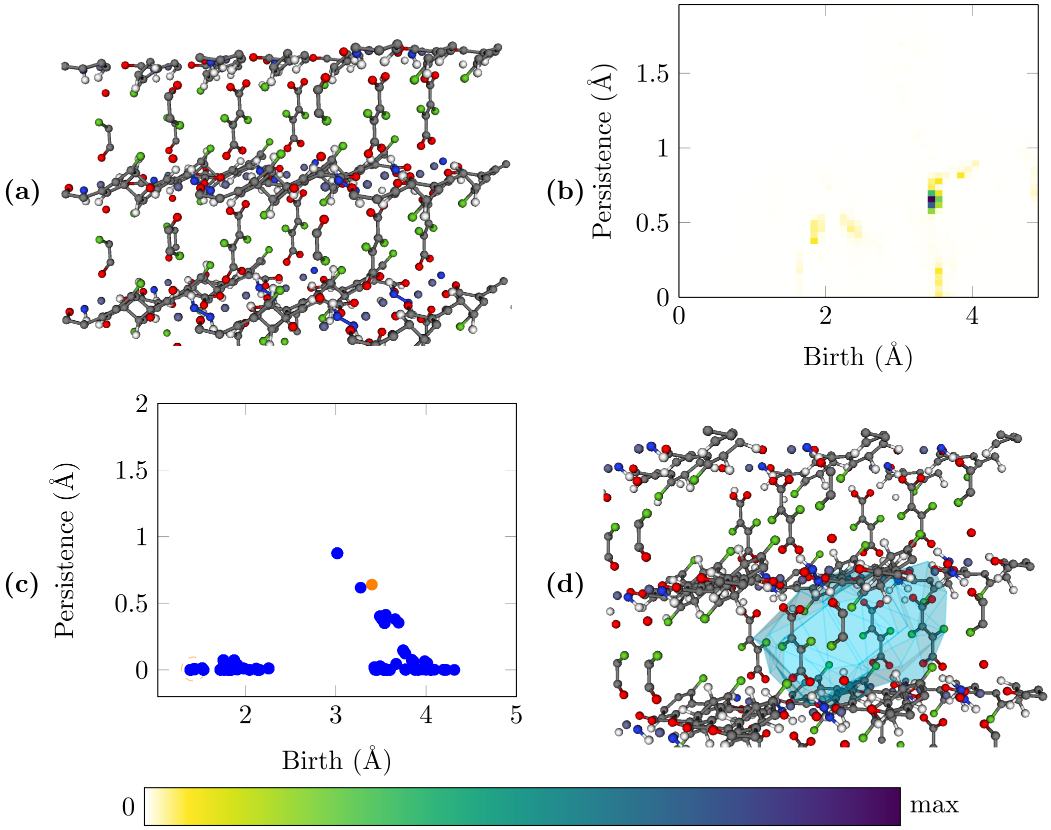

Figure 3: **Schematic outlining identification of a representative cycle in a crystal structure.** (a) The original crystal structure for MOF str-m3-o10-o15-pcu-sym.49 from the BW dataset. (b) The feature importance, shown as an image, for the 2D topology features for CO_2 adsorption at 0.15 bar. The color bar for this figure is shown at the bottom. (c) The 2D birth vs. persistence plot for str-m3-o10-o15-pcu-sym.49, with the (birth, persistence) point with the highest feature importance (as determined by the machine learning algorithm) in orange. The dashed lines around the orange point show the Gaussian spread factor. (d) The representative cycle for the closest point in the birth vs. persistence diagram to the orange point (specifically, the closest point that overlaps with the orange point) in (c). Figure created with VisIt 3.1.4 (`https://wci.llnl.gov/simulation/computer-codes/visit`).

1 High adsorption MOFs and composition

1.1 hMOFs dataset

Structure	Composition
hMOF-2279	(Zn, H, C, O, F)
hMOF-3250	(Zn, H, C, N, O, F)
hMOF-4	(Zn, H, C, O, F)
hMOF-673	(Zn, H, C, O, F)
hMOF-675	(Zn, H, C, O, F)
hMOF-1722	(Zn, H, C, O, F)
hMOF-2633	(Zn, H, C, O, F)
hMOF-2638	(Zn, H, C, O, F)
hMOF-469	(Zn, H, C, Cl, O)
hMOF-2287	(Zn, H, C, O, F)
hMOF-449	(Zn, H, C, O, F)
hMOF-7	(Zn, H, C, O, F)
hMOF-678	(Zn, H, C, O, F)
hMOF-1717	(Zn, H, C, O, F)
hMOF-953	(Zn, H, C, O)
hMOF-3152	(Zn, H, C, N, Cl, O)
hMOF-1721	(Zn, H, C, O, F)
hMOF-441	(Zn, H, C, O, F)
hMOF-1724	(Zn, H, C, O, F)
hMOF-3142	(Zn, H, C, N, O, F)

Table 1: hMOFs, 0.01 bar CO_2

Structure	Composition
hMOF-2279	(Zn, H, C, O, F)
hMOF-3250	(Zn, H, C, N, O, F)
hMOF-675	(Zn, H, C, O, F)
hMOF-4	(Zn, H, C, O, F)
hMOF-673	(Zn, H, C, O, F)
hMOF-2287	(Zn, H, C, O, F)
hMOF-1721	(Zn, H, C, O, F)
hMOF-16	(Zn, H, C, O, F)
hMOF-2283	(Zn, H, C, O, F)
hMOF-1724	(Zn, H, C, O, F)
hMOF-2633	(Zn, H, C, O, F)
hMOF-453	(Zn, H, C, O, F)
hMOF-2291	(Zn, H, C, O, F)
hMOF-958	(Zn, H, C, O, F)
hMOF-286	(Zn, H, C, O)
hMOF-1471	(Zn, H, C, Cl, O)
hMOF-1719	(Zn, H, C, O, F)
hMOF-441	(Zn, H, C, O, F)
hMOF-3246	(Zn, H, C, N, O, F)
hMOF-2638	(Zn, H, C, O, F)

Table 2: 0.05 bar CO_2

Structure	Composition
hMOF-2279	(Zn, H, C, O, F)
hMOF-3250	(Zn, H, C, N, O, F)
hMOF-675	(Zn, H, C, O, F)
hMOF-4	(Zn, H, C, O, F)
hMOF-673	(Zn, H, C, O, F)
hMOF-286	(Zn, H, C, O)
hMOF-13	(Zn, H, C, O, F)
hMOF-2291	(Zn, H, C, O, F)
hMOF-1721	(Zn, H, C, O, F)
hMOF-448	(Zn, H, C, O, F)
hMOF-16	(Zn, H, C, O, F)
hMOF-2287	(Zn, H, C, O, F)
hMOF-3018	(Zn, H, C, O)
hMOF-958	(Zn, H, C, O, F)
hMOF-2283	(Zn, H, C, O, F)
hMOF-1724	(Zn, H, C, O, F)
hMOF-1719	(Zn, H, C, O, F)
hMOF-2556	(Zn, H, C, O, F)
hMOF-2342	(Zn, H, C, O)
hMOF-3246	(Zn, H, C, N, O, F)

Table 3: 0.1 bar CO_2

Structure	Composition
hMOF-2342	(Zn, H, C, O)
hMOF-1595	(Zn, H, C, O, F)
hMOF-758	(Zn, H, C, O, F)
hMOF-498	(Zn, H, C, O)
hMOF-288	(Zn, H, C, O)
hMOF-756	(Zn, H, C, O, F)
hMOF-280	(Zn, H, C, O)
hMOF-760	(Zn, H, C, O, F)
hMOF-123	(Zn, H, C, O, F)
hMOF-3032	(Zn, H, C, O)
hMOF-3018	(Zn, H, C, O)
hMOF-282	(Zn, H, C, O)
hMOF-1507	(Zn, H, C, O)
hMOF-754	(Zn, H, C, O, F)
hMOF-1504	(Zn, H, C, O)
hMOF-751	(Zn, H, C, O, F)
hMOF-2294	(Zn, H, C, O, F)
hMOF-2290	(Zn, H, C, O, F)
hMOF-63	(Zn, H, C, O)
hMOF-3257	(Zn, H, C, N, O, F)

Table 4: 0.5 bar CO_2

Structure	Composition
hMOF-237	(Zn, H, C, O, F)
hMOF-3020	(Zn, H, C, O)
hMOF-234	(Zn, H, C, O, F)
hMOF-240	(Zn, H, C, O, F)
hMOF-2293	(Zn, H, C, O, F)
hMOF-228	(Zn, H, C, O, F)
hMOF-2289	(Zn, H, C, O, F)
hMOF-2277	(Zn, H, C, O, F)
hMOF-1440	(Zn, H, C, O, F)
hMOF-231	(Zn, H, C, O, F)
hMOF-2832	(Zn, H, C, O)
hMOF-2281	(Zn, H, C, O, F)
hMOF-2285	(Zn, H, C, O, F)
hMOF-1449	(Zn, H, C, O, F)
hMOF-436	(Zn, H, C, O, F)
hMOF-3248	(Zn, H, C, N, O, F)
hMOF-439	(Zn, H, C, O, F)
hMOF-3036	(Zn, H, C, O)
hMOF-3244	(Zn, H, C, N, O, F)
hMOF-834	(Zn, H, C, O, F)

Table 5: 2.5 bar CO_2

1.2 BW dataset

Structure	Composition
str_m2_o40_o40_fof_sym.19	(Cu, H, C, O, F)
str_m3_o40_o40_fof_sym.93	(Zn, H, C, Cl, O, F)
str_m5_o10_o29_sra_sym.189	(V, H, C, N, O, F)
str_m5_o16_o19_sra_sym.109	(V, H, C, O, F)
str_m3_o40_o40_fof_sym.76	(Zn, H, C, N, O, F)
str_m3_o1_o18_pcu_sym.101	(Zn, C, N, O, F)
str_m2_o1_o13_pcu_sym.157	(Cu, H, C, N, O, F)
str_m3_o10_o15_pcu_sym.49	(Zn, H, C, N, O, F)
str_m5_o16_o16_sra_sym.3	(V, H, C, O, F)
str_m2_o10_o29_pcu_sym.2	(Cu, H, C, N, O, F)
str_m3_o40_o40_fof_sym.73	(Zn, H, C, O)
str_m5_o1_o18_sra_sym.15	(V, H, C, O, F)
str_m5_o18_o18_sra_sym.27	(V, H, C, O, F)
str_m2_o10_o29_pcu_sym.88	(Cu, H, C, N, O, F)
str_m3_o10_o17_pcu_sym.151	(Zn, H, C, N, O)
str_m3_o12_o17_pcu_sym.61	(Zn, H, C, N, O, F)
str_m5_o17_o17_sra_sym.48	(V, H, C, Cl, O)
str_m5_o16_o16_sra_sym.11	(V, H, C, O, F)
str_m2_o10_o29_pcu_sym.211	(Cu, H, C, N, Cl, O)
str_m2_o10_o29_pcu_sym.139	(Cu, H, C, N, O)

Table 6: BW, 0.15 bar CO_2

Structure	Composition
str_m7_o3_o3_bcu_sym.53	(Ni, H, C, N, O)
str_m3_o11_o29_nbo_sym.81	(Zn, H, C, N, O, F)
str_m3_o34_o35_pts_sym.67	(Zn, H, C, O, F)
str_m3_o19_o29_nbo_sym.12	(Zn, H, C, N, O, F)
str_m3_o7_o25_pcu_sym.31	(Zn, H, C, N, O, F)
str_m2_o2_o6_pcu_sym.58	(Cu, H, C, N, O, F)
str_m3_o2_o28_pcu_sym.144	(Zn, H, C, N, O, F)
str_m3_o3_o26_pcu_sym.176	(Zn, H, C, N, O, F)
str_m3_o2_o5_nbo_sym.40	(Zn, H, C, O, F)
str_m3_o2_o29_pcu_sym.75	(Zn, H, C, N, Cl, O)
str_m3_o8_o20_pcu_sym.237	(Zn, H, C, N, O, F)
str_m1_o19_o29_pcu_sym.37	(Zn, H, C, N, O, F)
str_m3_o3_o23_pcu_sym.5	(Zn, H, C, N, O, F)
str_m3_o18_o29_pcu_sym.124	(Zn, H, C, N, O, F)
str_m3_o21_o28_pcu_sym.137	(Zn, H, C, N, O)
str_m3_o25_o29_pcu_sym.49	(Zn, H, C, N, O, F)
str_m3_o6_o25_pcu_sym.247	(Zn, H, C, N, O, F)
str_m3_o3_o17_pcu_sym.38	(Zn, H, C, N, O, F)
str_m2_o26_o27_pcu_sym.21	(Cu, H, C, N, O, F)
str_m2_o6_o18_pcu_sym.214	(Cu, H, C, N, O, F)

Table 7: BW, 16 bar CO_2

Structure	Composition
str_m5_o18_o18_sra_sym.23	(V, H, C, O)
str_m5_o18_o18_sra_sym.87	(V, H, C, N, O)
str_m5_o18_o18_sra_sym.1	(V, H, C, O)
str_m2_o5_o17_pcu_sym.16	(Cu, H, C, N, O)
str_m5_o18_o18_sra_sym.57	(V, H, C, O)
str_m5_o18_o18_sra_sym.38	(V, H, C, O)
str_m5_o18_o18_sra_sym.54	(V, H, C, O)
str_m3_o10_o15_pcu_sym.1	(Zn, H, C, N, O)
str_m5_o18_o18_sra_sym.11	(V, H, C, O, F)
str_m3_o5_o17_pcu_sym.24	(Zn, H, C, N, O)
str_m2_o10_o29_pcu_sym.144	(Cu, H, C, N, O)
str_m2_o10_o29_pcu_sym.3	(Cu, H, C, N, O)
str_m2_o10_o29_pcu_sym.20	(Cu, H, C, N, O, F)
str_m2_o10_o29_pcu_sym.146	(Cu, H, C, N, O)
str_m3_o10_o29_pcu_sym.87	(Zn, H, C, N, O)
str_m2_o10_o29_pcu_sym.107	(Cu, H, C, N, O)
str_m2_o10_o29_pcu_sym.143	(Cu, H, C, N, O)
str_m2_o10_o29_pcu_sym.221	(Cu, H, C, N, O)
str_m2_o10_o29_pcu_sym.138	(Cu, H, C, N, O)
str_m2_o10_o29_pcu_sym.1	(Cu, H, C, N, O)

Table 8: BW, 5.8 bar CH_4

Structure	Composition
str_m3_o40_o41_fof_sym.127	(Zn, H, C, O)
str_m2_o41_o41_fof_sym.42	(Cu, H, C, O)
str_m2_o41_o41_fof_sym.24	(Cu, H, C, O)
str_m3_o40_o40_fof_sym.22	(Zn, H, C, O)
str_m2_o40_o41_fof_sym.52	(Cu, H, C, O)
str_m3_o40_o41_fof_sym.14	(Zn, H, C, N, O)
str_m2_o41_o41_fof_sym.22	(Cu, H, C, O)
str_m3_o41_o41_fof_sym.1	(Zn, H, C, O)
str_m3_o40_o41_fof_sym.27	(Zn, H, C, N, O)
str_m3_o40_o41_fof_sym.52	(Zn, H, C, N, O)
str_m3_o40_o41_fof_sym.3	(Zn, H, C, O, F)
str_m3_o41_o41_fof_sym.62	(Zn, H, C, O)
str_m3_o40_o41_fof_sym.45	(Zn, H, C, O, F)
str_m3_o40_o41_fof_sym.228	(Zn, H, C, O)
str_m3_o17_o17_pcu_sym.99	(Zn, H, C, N, O)
str_m3_o40_o41_fof_sym.10	(Zn, H, C, O)
str_m5_o18_o19_sra_sym.27	(V, H, C, O)
str_m2_o40_o41_fof_sym.7	(Cu, H, C, Cl, O)
str_m3_o41_o41_fof_sym.82	(Zn, H, C, O)
str_m2_o41_o41_fof_sym.46	(Cu, H, C, N, O)

Table 9: BW, 65 bar CH_4

Structure	Composition
str_m4_o4_o5_acs_sym.8	(Cr, H, C, O, F)
str_m4_o14_o14_acs_sym.24	(Cr, H, C, O, F)
str_m4_o1_o22_acs_sym.197	(Cr, H, C, N, Cl, O)
str_m4_o1_o22_acs_sym.94	(Cr, C, Cl, O, F)
str_m4_o1_o1_acs_sym.10	(Cr, H, C, N, O)
str_m4_o14_o14_acs_sym.119	(Cr, H, C, O, F)
str_m4_o4_o15_acs_sym.125	(Cr, H, C, O, F)
str_m4_o1_o15_acs_sym.56	(Cr, H, C, N, O, F)
str_m4_o1_o1_acs_sym.46	(Cr, H, C, Cl, O)
str_m4_o11_o14_acs_sym.79	(Cr, H, C, S, O, F)
str_m4_o1_o24_acs_sym.165	(Cr, H, C, Br, Cl, O)
str_m4_o1_o14_acs_sym.68	(Cr, H, C, N, O)
str_m4_o1_o24_acs_sym.96	(Cr, H, C, N, Cl, O)
str_m4_o12_o15_acs_sym.49	(Cr, H, C, N, O)
str_m4_o1_o24_acs_sym.25	(Cr, C, Cl, O, F)
str_m4_o4_o15_acs_sym.65	(Cr, H, C, O, F)
str_m4_o1_o14_acs_sym.145	(Cr, H, C, Br, N, O)
str_m5_o1_o18_sra_sym.35	(V, H, C, O, F)
str_m4_o12_o15_acs_sym.129	(Cr, H, C, Cl, O, F)
str_m4_o1_o22_acs_sym.179	(Cr, H, C, Cl, O, F)

Table 10: BW, log(k_H) CO_2

Structure	Composition
str_m4_o17_o17_acs_sym.152	(Cr, H, C, O)
str_m4_o17_o17_acs_sym.14	(Cr, H, C, O)
str_m4_o17_o17_acs_sym.13	(Cr, H, C, O)
str_m4_o17_o17_acs_sym.133	(Cr, H, C, Cl, O)
str_m4_o17_o17_acs_sym.99	(Cr, H, C, O)
str_m5_o5_o13_sra_sym.60	(V, H, C, O)
str_m4_o17_o17_acs_sym.82	(Cr, H, C, O)
str_m4_o17_o17_acs_sym.69	(Cr, H, C, O)
str_m4_o17_o17_acs_sym.122	(Cr, H, C, N, O)
str_m5_o13_o18_sra_sym.4	(V, H, C, O)
str_m4_o17_o17_acs_sym.171	(Cr, H, C, O)
str_m4_o17_o17_acs_sym.108	(Cr, H, C, O)
str_m4_o17_o17_acs_sym.56	(Cr, H, C, O)
str_m3_o10_o15_pcu_sym.23	(Zn, H, C, N, O)
str_m4_o17_o17_acs_sym.16	(Cr, H, C, O)
str_m5_o5_o2_sra_sym.49	(V, H, C, O)
str_m4_o17_o17_acs_sym.150	(Cr, H, C, S, O)
str_m4_o17_o17_acs_sym.114	(Cr, H, C, O)
str_m4_o17_o17_acs_sym.123	(Cr, H, C, O)
str_m2_o10_o17_pcu_sym.220	(Cu, H, C, N, O)

Table 11: BW, log(k_H) CH_4

1.3 CoREMOF dataset

Structure	Composition
KUXSAZ_clean	(Fe, H, C, N, O)
TOXMUQ_clean	(Al, H, C, O)
acs.cgd.5b01632_ZAHKOL1438120_clean	(Cu, H, C, N, O)
TOXNIF_clean	(Al, H, C, O)
IMUVES_clean	(In, C, O)
XUPSAE_clean	(Al, H, C, O)
TOXNEB_clean	(Al, H, C, O)
FEVNOL_clean	(Cu, C, I, N)
HITXUE_clean	(Al, P, O)
COQNIF_clean	(Mn, Al, P, O)
SAPJEZ_clean	(Al, P, O)
YIGHIG_clean	(Cu, H, C, N)
PANRUS01_clean	(Al, P, O)
MUTGUD_clean	(Ag, H, C, N)
VURNED04_clean	(Zn, H, C, N, O)
YEMTIV_clean	(Cu, H, C, N)
TAKTIL_clean	(Ga, H, C, O)
BICPOT_clean	(Al, P, O, F)
KAVXAI_clean	(Co, H, C, N, O)
VURNED_clean	(Zn, H, C, N, O)

Table 12: CoREMOF, 5.8 bar CH_4

Structure	Composition
YEMTER_clean	(Cu, H, C, N)
VURNED04_clean	(Zn, H, C, N, O)
VURNED_clean	(Zn, H, C, N, O)
WAFKIY_clean	(Zn, H, C, N, O)
QABKIQ_clean	(Li, P, C, N, F)
MOYZIK_clean	(V, Zn, H, C, N, O)
VURNED02_clean	(Zn, H, C, N, O)
VURNED01_clean	(Zn, H, C, N, O)
MOKVOZ_clean	(Cu, H, C, O)
c6ce00465b_c6ce00465b2_clean	(In, H, C, O)
LEQCAN_clean	(Cu, H, C, O)
IMUVES_clean	(In, C, O)
acs.inorgchem.6b00661_ic6b00661_si_002_clean	(Al, C, O)
LAQNUP_clean	(Cu, H, C, O)
KAVXAI_clean	(Co, H, C, N, O)
IGORUS_clean	(Zn, H, C, N, O)
CESFIQ_clean	(Cu, H, C, O)
acs.cgd.5b01632_ZAHKOL1438120_clean	(Cu, H, C, N, O)
MUKQUG_clean	(Cu, H, C, O)
LAQNOJ_clean	(Cu, H, C, O)

Table 13: CoREMOF, 65 bar CH_4

Structure	Composition
ZITWIK01_clean	(Cd, N)
ZISXEG_clean	(Cu, H, C, I, N)
IPEBAI_clean	(Cu, H, C, I, N)
MISQIQ_clean	(Al, P, H, O, F)
MISQIQ02_clean	(Al, P, H, O, F)
MISQIQ04_clean	(Al, P, H, O, F)
VOKJIQ_clean	(Al, P, H, O)
MISQIQ06_clean	(Al, P, H, O, F)
MISQIQ07_clean	(Al, P, H, O, F)
MISQIQ01_clean	(Al, P, H, O, F)
MISQIQ05_clean	(Al, P, H, O, F)
MISQIQ03_clean	(Al, P, H, O, F)
GOMPOO_clean	(Al, P, H, O)
ECIYEU_clean	(Al, P, H, O)
XULFOA_clean	(Cu, H, C, N, O)
VABLUG_clean	(Ag, H, C, N)
CAHQOS_clean	(Al, P, O)
FIJYED_clean	(Al, P, O)
MORZID_clean	(Al, P, O)
QOLVET_clean	(Al, P, O)

Table 15: log(k_H) CH_4

Structure	Composition
DIVNIH_clean	(Nd, C, S, O)
XEHFUL01_clean	(Y, C, O)
HOZDIL_clean	(Al, P, H, C, O)
YEZKIZ_clean	(Cr, Ni, H, C, N, O)
DIPMAS_clean	(Ce, P, C, O)
YAWKOX_clean	(Pr, H, C, O)
ETUWIA_clean	(Y, C, O)
TAGSEB_clean	(V, Cu, H, C, N, O)
ELIKAM_clean	(Pr, H, C, O)
CIKCOQ_clean	(Cd, H, C, N, O)
VOKJIQ_clean	(Al, P, H, O)
KIBDEF_clean	(Eu, H, C, O)
c5nj02907d_c5nj02907d2_clean	(V, H, C, N, O)
WAPTAL_clean	(Nd, As, P, H, W, C, O)
BUWMAJ_clean	(Nd, H, C, N, O)
XOCWET_clean	(La, U, H, C, O)
FATKUI_clean	(Ce, H, C, N, O)
DATHAJ_clean	(Nd, H, C, N, O)
PUMGIP_clean	(V, Cd, H, C, N, O)
VIWMAR_clean	(Nd, Cu, H, C, O)

Table 14: log(k_H) CO_2
